# Severe Impaction of the Primary Mandibular Second Molar Accompanied by Displacement of the Permanent Second Premolar

**DOI:** 10.1155/2015/582462

**Published:** 2015-02-25

**Authors:** Junko Matsuyama, Shoko Kinoshita-Kawano, Sachiko Hayashi-Sakai, Tomoe Mitomi, Tomiko Sano-Asahito

**Affiliations:** Division of Pediatric Dentistry, Graduate School of Medical and Dental Sciences, Niigata University, 2-5274 Gakkocho-dori, Chuo-ku, Niigata 951-8514, Japan

## Abstract

Tooth impaction is defined as any tooth that fails to erupt into a normal functional position and remains unerupted beyond the time at which it should normally erupt. Reports of impaction and eruption failure in primary teeth are relatively rare compared to permanent teeth. We report 2 rare cases where the second premolar was located on the occlusal side of the impacted mandibular second primary molar. In the first case, the succedaneous permanent tooth erupted after extraction of the primary tooth, fenestration, and traction. In the second case, the succedaneous permanent tooth erupted without fenestration or traction. Although the etiology of the tooth displacement was unknown in both cases, inhibition of the eruptive movement of the primary molar may have been associated with displacement of the succedaneous permanent premolar.

## 1. Introduction

Tooth impaction is defined as any tooth that fails to erupt into a normal functional position and remains unerupted in the jaw beyond the time when it should normally erupt [1]. Reports of impaction and eruption failure in primary teeth are relatively rare compared to permanent teeth [1, 2]. Among primary tooth impaction cases, second primary molars are most frequently affected, followed by primary central incisors [2, 3]. Since impaction of a primary tooth with displacement of the succedaneous permanent tooth might disturb the growth of the permanent dental arch, detection and treatment of impacted primary teeth are essential. In this study, we report 2 rare cases of severe impaction of the mandibular second primary molar where the succedaneous second premolar was located over the impacted second primary molar.

## 2. Case Presentations

### 2.1. Case  1

**Figure 1:**
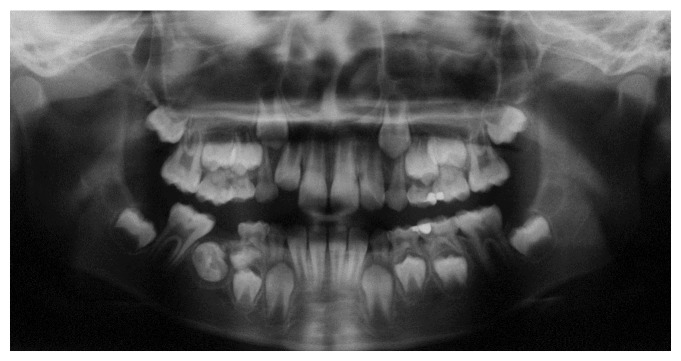
Initial panoramic radiograph: case  1. Complete impaction and mesiolingual inclination of the mandibular right second primary molar in the mandibular bone.

**Figure 2:**
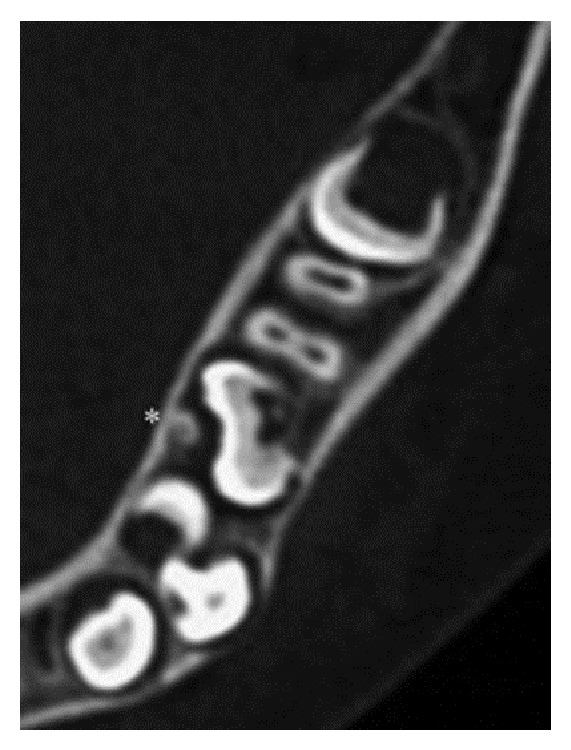
Axial computed tomography image: case  1. A small calcium deposit (∗) located lingual to the mandibular right second primary molar.

A 7-year-10-month-old female patient was referred to the Pediatric Dentistry Department of Niigata University Medical and Dental Hospital by a dentist at another clinic. The chief complaint was the eruption failure of the mandibular right second primary molar. Family and medical history were unremarkable. Intraoral examination confirmed absence of the mandibular right second primary molar. No redness or swelling of the overlying gingiva was apparent. A panoramic radiograph revealed complete impaction and mesiolingual inclination of the mandibular right second primary molar in the mandibular bone (Figure 1). The permanent mandibular right second premolar was mesioversed and located between the apical side of the mandibular right first primary molar and the occlusal side of the second primary molar. Computed tomography showed a small calcium deposit located lingual to the impacted mandibular right second primary molar (Figure 2).

**Figure 3:**
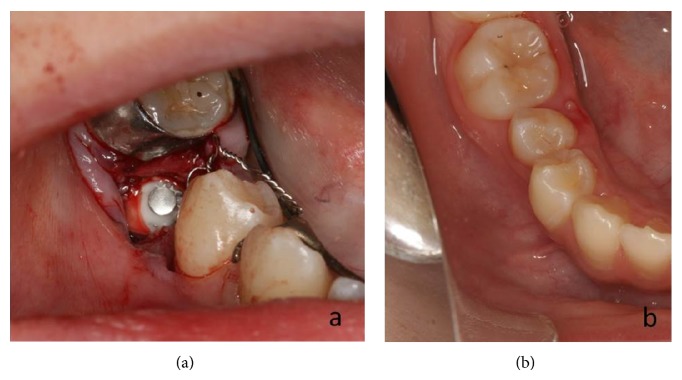
Intraoral photographs: case  1. (a) Fenestration was performed and traction applied to the permanent second premolar at age of 10 years and 2 months. (b) The permanent mandibular right second premolar has erupted into occlusion 14 months after traction at age of 11 years and 4 months.

The impacted mandibular right second primary molar was extracted and the calcium deposit eliminated. The patient was followed and underwent periodic radiographic examinations. The permanent mandibular right second premolar failed to erupt, although it showed slight movement distally. Therefore, when the patient was 10 years and 2 months old (2 years and 4 months after surgical treatment), fenestration was performed, followed by immediate traction of the unerupted tooth (Figure 3(a)). Six months after fenestration and traction, part of the tooth crown could be observed in the oral cavity. Fourteen months after traction, the permanent mandibular right second premolar erupted into occlusion (Figure 3(b)).

### 2.2. Case  2

**Figure 4:**
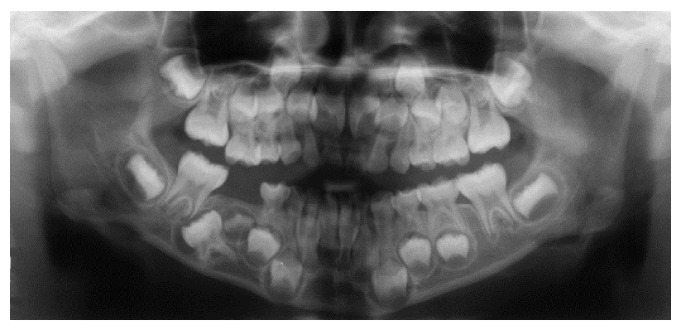
Initial panoramic radiograph: case  2. Complete impaction and distal inclination of the mandibular right second primary molar in the mandibular bone.

**Figure 5:**
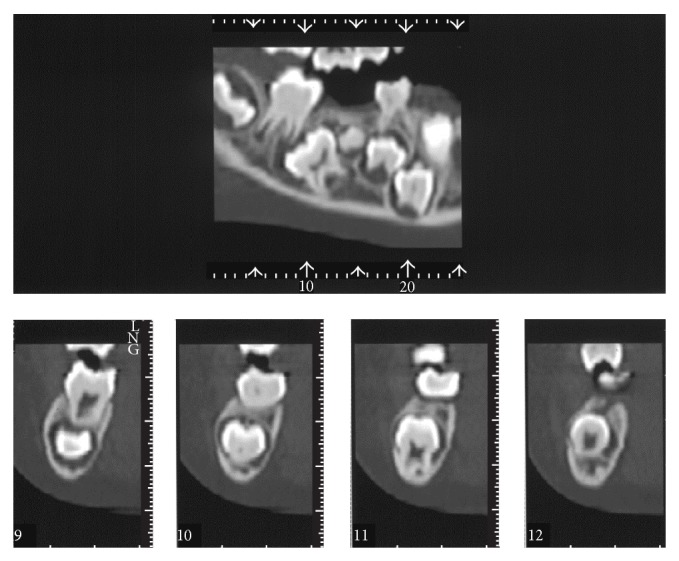
Sagittal reconstruction computed tomography: case  2. The root apex of the impacted primary molar is extremely close to the mandibular canal and mandibular plane.

**Figure 6:**
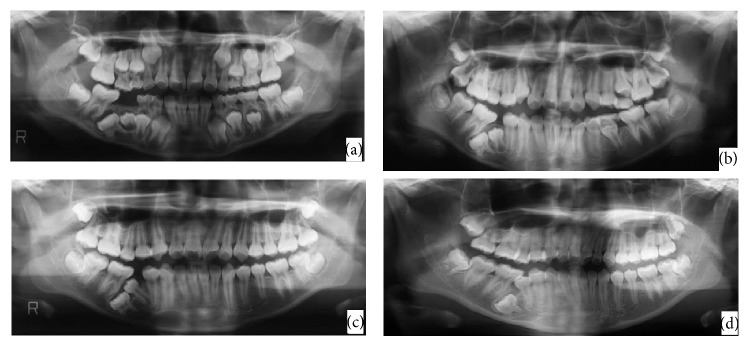
Panoramic radiographs showing growth changes of the dental germ: case  2. The formation stages of the impacted permanent tooth were approximately (a) Ri at age of 9 years and 2 months, (b) R1/2 at age of 11 years and 0 months, and (c) R3/4 at age of 12 years and 6 months. (d) At age of 16 years and 7 months, most of the tooth crown of the second permanent premolar had erupted, but the second primary molar remained impacted.

A 6-year-4-month-old female patient presented to Pediatric Dentistry Department of Niigata University Medical and Dental Hospital with the chief complaint of a missing mandibular right second primary molar that was pointed out during a school dental health examination. Family and medical history were unremarkable. Intraoral examination confirmed absence of the mandibular right second primary molar. A panoramic radiograph revealed complete impaction and distal inclination of the mandibular right second primary molar in the mandibular bone (Figure 4). The permanent mandibular right second premolar was located mesial to the second primary molar. Sagittal reconstruction computed tomography (CT) revealed that the root apex of the impacted primary molar was extremely close to the mandibular canal and mandibular plane (Figure 5). Furthermore, the impacted second primary molar was surrounded by X-ray permeable regions with clear boundaries in panoramic radiograph and CT. Therefore, it was decided not to extract the impacted primary second molar to avoid injuring the mandibular canal and neighboring dental germs. The patient was followed and underwent periodic radiographic examinations. The growth changes of the dental germ revealed on the panoramic radiographs are shown in Figure 6. According to a Moorrees et al.'s [4] classification of tooth formation, the degree of formation of the impacted permanent second premolar was approximately Ri (initial root formation stage) at age of 9 years and 2 months (Figure 6(a)), approximately R1/2 at 11 years (Figure 6(b)), and approximately R3/4 at 12 years and 6 months (Figure 6(c)). At the age of 15 years and 5 months (9 years and 1 month after the first visit), the permanent mandibular right second premolar could be partially observed. At age of 16 years and 7 months (11 years and 3 months after the first visit), most of the tooth crown of the second permanent premolar had erupted, but the second primary molar remained impacted (Figure 6(d)).

## 3. Discussion

In the present cases, the most characteristic finding was the location of the second premolar on the occlusal side of the impacted mandibular second primary molar. To date, there have been relatively few reports of impaction and tooth eruption failure in primary teeth compared to permanent teeth [2]. Kjær et al. [5] evaluated radiographs of arrested eruption of primary molars and found that the permanent successors were located on the occlusal side of the unerupted primary molar in 4 of 29 cases. A survey of the literature revealed only a few reported cases [6–11] in which a permanent tooth germ was located over the impacted primary molar.

Tooth eruption takes place in several stages [12]. Early in the preeruptive phase, the successional permanent teeth develop lingual to and near the occlusal level of their primary predecessors. At the end of this phase, the premolars are located under the roots of the primary molars. The change in position of the tooth germ is primarily not the result of apical movement of the permanent tooth germs, but of eruption of the primary tooth and the coincident increase in the height of supporting tissues [13]. During development, the dental germ of the lower second premolar originates from the successional tooth band at the lingual side of the dental germ of the mandibular second primary molar [14]. As a cause of the positional reversal, we inferred that the inhibition of preeruptive movement of the primary molar may have been associated with displacement of the succedaneous permanent premolar.

The etiology of tooth impaction includes systemic and local factors such as dental germ abnormality, eruption cyst, odontoma, tooth displacement, ankylosis, gingival hyperplasia, and eruption space deficiency [1]. In the present cases, there were no relevant considerations in the family or medical history and the contralateral second primary molar erupted normally, and therefore systemic etiological factors can be ruled out. Similar to supernumerary teeth and odontoma, calcium deposition is considered one of the causes of tooth impaction. The cause of impaction of the primary tooth in case  2 remains unknown. In cases where an etiological factor such as a calcium deposition is clear, the first choice of treatment for the impacted tooth should be elimination of the factor. In cases without such a clear etiological factor, where the eruption space of the second primary molar has been procured, the first choice of treatment is fenestration and traction of the second primary molar.

In case  1, unfortunately histopathological findings of calcium deposit were not available, since consent on the pathological examination was not obtained from the patient. However, the radiographic features showed a well-defined smooth border and a tooth-like appearance of radiopaque structure that typical images of odontoma [15]; thus the calcium deposit was suggested to be odontoma. Furthermore, the succedaneous permanent tooth erupted after extraction of the impacted primary second molar, elimination of the calcium deposit, fenestration, and traction.

On the other hand, in case  2, second primary molar was not extracted and remained impacted deep down in the mandible, because the root apex was extremely close to the mandibular canal and mandibular plane. If the follicular space of impacted teeth exceeded 5 mm a dentigerous cyst was more likely [15]; however in this case the follicular space was 2 to 3 mm and the structure was considered to be dental follicle. Furthermore, on CT images, Hounsfield Unit (HU) of X-ray permeable regions surrounding impacted second primary molar were approximately 60 HU, which were similar to values for sound permanent mandibular third molars (approximately 50 HU). Thus, the structure was unlikely to be cysts, because the density was higher than that in cystic regions (about 20 HU) [16]. However, the presence of impacted teeth may lead to clinical problems such as root resorption of permanent teeth, cyst formation, and malocclusion; thus a long-term observation is necessary.

In cases where the permanent first molar has erupted and the eruption space for the impacted second primary molar and/or premolar is insufficient because of mesial inclination and mesial displacement of the permanent first molar, distalization of the first molar is required to procure sufficient eruption space for the deciduous molar and/or premolar. It is important that pediatric dentists detect the impaction of primary molars during primary dentition to prevent disturbing the complete and sound eruption of permanent dentition and avoid treatment complications. Delayed detection and treatment of primary tooth impaction might cause delayed eruption, impaction, and displacement of the succedaneous permanent tooth.

## 4. Conclusions

Impaction and eruption failure of primary teeth might be associated with a disturbance of the permanent successors. Unfortunately, in case  2, second primary molar remained impacted deep down in the mandible, but orthodontic traction or extraction might be applied if impaction was discovered early. It is important that pediatric dentists detect the impaction of primary molars during primary dentition to prevent disturbing the complete and sound eruption of permanent dentition and avoid treatment complications. Furthermore, a long-term observation is necessary until the permanent successors erupt.
